# Motivational interviewing for low mood and adjustment early after stroke: a feasibility randomised trial

**DOI:** 10.1186/s40814-018-0343-z

**Published:** 2018-09-25

**Authors:** Kulsum Patel, Caroline L Watkins, Chris J Sutton, Emma-Joy Holland, Valerio Benedetto, Malcolm F Auton, David Barer, Kausik Chatterjee, Catherine E Lightbody

**Affiliations:** 1 Faculty of Health and Wellbeing, University of Central Lancashire, Preston, UK; 2 Faculty of Health Sciences, Australian Catholic University, Sydney, Australia; 3 Faculty of Biology, Medicine and Health, University of Manchester, Manchester, UK; 4 Faculty of Medical Sciences, Newcastle University, Newcastle upon Tyne, UK; 5 Countess of Chester Hospital NHS Foundation Trust, Chester, UK

**Keywords:** Feasibility, Motivational interviewing, Stroke, Psychological adjustment

## Abstract

**Background:**

Management of psychological adjustment and low mood after stroke can result in positive health outcomes. We have adapted a talk-based therapy, motivational interviewing (MI), and shown it to be potentially effective for managing low mood and supporting psychological adjustment post-stroke in a single-centre trial. In the current study, we aimed to explore the feasibility of delivering MI using clinical stroke team members, and using an attention control (AC), to inform the protocol for a future definitive trial.

**Methods:**

This parallel two-arm feasibility trial took place in north-west England. Recruitment occurred between December 2012 and November 2013. Participants were stroke patients aged 18 years or over, who were medically stable, had no severe communication problems, and were residents of the hospital catchment. Randomisation was to MI or AC, and was conducted by a researcher not involved in recruitment using opaque sealed envelopes. The main outcome measures were descriptions of study feasibility (recruitment/retention rates, MI delivery by clinical staff, use of AC) and acceptability (through qualitative interviews and completion of study measures), and fidelity to MI and AC (through review of session audio-recordings). Information was also collected on participants’ mood, quality of life, adjustment, and resource-use.

**Results:**

Over 12 months, 461 patients were screened, 124 were screened eligible, and 49 were randomised: 23 to MI, 26 to AC. At 3 months, 13 MI and 18 AC participants completed the follow-up assessment (63% retention). This was less than expected based on our original trial. An AC was successfully implemented. Alternative approaches would be required to ensure the feasibility of clinical staff delivering MI. The study measures, MI, and AC interventions were considered acceptable, and there was good fidelity to the interventions. There were no adverse events related to study participation.

**Conclusions:**

It was possible to recruit and retain participants, train clinical staff to deliver MI, and implement an appropriate AC. Changes would be necessary to conduct a future multi-centre trial, including: assuming a recruitment rate lower than that in the current study; implementing more strategies to increase participant retention; and considering alternative clinical staff groups to undertake the delivery of MI and AC.

**Trial registration:**

ISRCTN study ID: ISRCTN55624892

**Trial funding:**

Northern Stroke Research Fund

## Background

Stroke is a leading cause of adult disability and occurs in over 150,000 people each year in the UK [1]. Psychological and mood issues are common after stroke, with one in every three stroke survivors experiencing depression [2]. Depression following stroke is an independent predictor of poor recovery, including a lower quality of life and more severe disability [3]. Depressed stroke survivors lack motivation to participate in rehabilitation, engage less in leisure and social activities, and are more likely to die than non-depressed stroke survivors [4]. Preventing and treating depression after stroke could reduce the burden to individuals and improve outcome. However, psychological support following stroke is lacking, with stroke survivors reporting this as an unmet need [5]. There is also little conclusive evidence on the management of psychological issues following stroke, in terms of preventing or treating depression [6, 7].

The findings of pooled analyses of previous studies exploring the effect of psychotherapy on the prevention of depression indicated a small but significant benefit [6]. This effect became non-significant with the removal of the results of our previous trial [8]. In our study, we investigated motivational interviewing (MI) for supporting adjustment after stroke. MI is an established talking therapy, traditionally used in the context of changing problematic behaviour. It is a person-centred, directive but constructive talking therapy. Using specific MI person-centred techniques, the MI therapist increases awareness and the importance of change through sensitively amplifying the discrepancy between current issues and the person’s goals or personal values. Confidence is then built through supporting self-efficacy, enabling the person to develop motivation and readiness to change. For our study, MI was adapted specifically so that it could be delivered to stroke survivors early after their stroke to develop motivation to engage in the rehabilitation process, to facilitate adjustment to having had a stroke, and to promote a sense of self-efficacy in managing life after stroke. At 3 months after stroke, those who received up to four sessions of MI in addition to usual care (*n* = 204) were less likely to have low mood than those who received only usual care (*n* = 207) [8]; this effect was maintained at 12 months after stroke [9].

Although our findings suggested that MI has the potential to be used to effectively prevent or treat depression following stroke, there were some limitations. The comparator group consisted of usual care and it may be that the effect was due, at least partially, to the additional attention received by the intervention group rather than MI itself. By providing participants in a control group with social attention of similar duration and intensity to the MI therapy, any difference between the two groups should be attributable to the specific nature of the input. Therefore, there is a need to identify and choose an attention control which will be a more appropriate comparator to MI to account for the additional attention received by those in the intervention arm. Another limitation of our previous study is that the intervention was delivered by MI therapists who were members of the research team and who were trained and supervised externally to the clinical setting. Consequently, there is no indication of how the intervention might be delivered and how training and supervision of MI therapists might occur as part of practice.

Our aim was to explore the feasibility of delivering MI using members of the clinical team, and using an attention control (AC), to inform the protocol for a future definitive trial.

### Objectives

- Estimate the recruitment and 3-month retention rates of participants
- Estimate the completeness of data capture in study measures
- Explore acceptability of the MI, AC, and study processes and materials to staff and patients
- Explore the implementation of each intervention and associated challenges, and understand the contextual factors influencing implementation
- Describe the recruitment, training, and retention of staff delivering the intervention
- Estimate fidelity to MI and AC interventions

## Methods

### Study design

This was a mixed-methods single-centre feasibility study, incorporating a non-blinded parallel-group randomised controlled feasibility trial (MI vs. AC, allocation ratio 1:1), and interviews with staff and participants. Ethical approval was obtained from the local research ethics committee.

### Setting

The study was conducted in one acute stroke unit within a hospital serving a predominantly urban population in the North West of England.

### Study staff

Six therapy assistants were identified from the multidisciplinary stroke team to deliver the MI or AC intervention, and backfill was provided. There were no specific criteria for the selection of the study staff: the therapy team manager identified the therapy assistants based on their own judgement of who would be most appropriate for the roles. All six staff were given basic training by members of the research team. Basic training comprised two full-day sessions delivered in person and covered background information on stroke, and practical information for conducting research (research governance guidelines, confidentiality, ward procedures, home visiting procedures, safety guidelines, and reporting adverse events or incidents). After basic training, staff were randomised to deliver either MI or AC (three therapy assistants to each).

### Training–MI

MI-specific training was delivered by MI therapists from our previous study. Training comprised an introductory 1-day workshop incorporating the theory behind MI, psychological mechanisms that effect change, and familiarisation with our MI manual developed by the research team prior to this study. This was followed by practice MI sessions among the three MI therapists, which were video-recorded for therapists to reflect on their skills and for trainers to provide feedback. The MI therapists then undertook at least 10 practice sessions with volunteer patients until confidence and threshold competency (assessed with the Motivational Interviewing Treatment Integrity Code (MITI) [10]) were achieved. The initial sessions with volunteer patients were observed by the trainers in person and later practice sessions were carried out by the therapist alone. Ongoing supervision was provided by the trainers, both face-to-face and remotely via telephone and email, and was scheduled to occur once a month, but therapists could contact the trainers at any point in between scheduled supervision meetings for support. Competency was monitored by the trainers through review of audio-recordings of sessions throughout the study period.

### Training–AC

AC-specific training was based on the AC intervention used in the Accessing Communication Therapy in the North West (ACTNoW) study [11] and was delivered by the AC monitor in the ACTNoW study. Training comprised an introductory 1-day workshop, followed by practice AC sessions among the 3 AC visitors and then with at least 10 volunteer patients until competence and confidence in delivering the AC was achieved, as determined by the AC monitor through review of audio-recordings of practice sessions. Ongoing supervision was provided by the AC monitor and the study’s research team, and competency was monitored through audio-recordings throughout the study period.

### Intervention–MI

The MI intervention comprised four 1-h sessions, structured so that the first was an introductory session for building rapport, where the therapist set the agenda and the patient talked about their adjustment to stroke and current concerns. The second and third sessions involved working through patients’ concerns. The final session was for winding down and was used to explore unresolved issues from previous sessions, review the sessions as a whole, and terminate the intervention in a mutually safe and satisfactory manner. The application of MI principles for this intervention has been described previously [8]. MI therapists also completed the Working Alliance Inventory (WAI) [12], a measure of therapeutic alliance, after each session.

### Intervention–AC

The format of the AC was designed to reflect the format of the MI intervention such that the only real difference between the two was the active component of MI. The content of the AC was multi-faceted and tailored to individual needs, interests, state of health, and abilities. The AC was structured to incorporate three stages over four 1-h sessions. The first session was an introductory session for building rapport. The second and third sessions were for regular contact. Sessions aimed to be participant-led through general conversation but AC visitors had access to basic materials (e.g. playing cards, newspapers) to suggest appropriate activities (i.e. activities not focused on mood). The final session was for winding down and bringing the AC sessions to an end.

### Participants

Consecutive patients admitted to the acute stroke unit with suspected stroke between December 2012 and November 2013 were screened for eligibility within 5 days of stroke onset. Patients were eligible if they were aged 18 years or over, had a diagnosis of stroke, were medically stable, had no severe communication problems or lack of capacity to consent (based on an observational communication checklist devised specifically for this study and clinical staff judgement), and lived within the hospital catchment. For patients who were initially ineligible (not medically stable within 5 days of stroke onset), screening was repeated weekly within hospital for up to 4 weeks post-stroke onset. All participants provided written informed consent. Screening and consent procedures were undertaken by a research nurse or therapy assistant.

### Sample size

Based on the 400 patients with stroke presenting to the acute stroke unit annually, we estimated that we would recruit approximately 118 participants over a 1-year recruitment period, assuming the 50% eligibility rate and 59% consent rate among those eligible from our previous trial [8]. Based on this consent rate (and 200 eligible patients), this would enable estimation of the true rate to within ± 6.8% and estimation of the retention rate to within ± 7.2%, assuming the true rate to be 80% (or greater), each with 95% confidence.

### Baseline measures

A research nurse or therapy assistant carried out baseline assessments once the participant had consented. The following participant characteristics were collected: age, sex, stroke side, past medical history of psychological problems, mental health services input, and antidepressant use (from medical notes). The following baseline measures were collected: cognition (Addenbrooke’s Cognitive Examination Revised (ACE-R) [13]), mood (General Health Questionnaire 12 item (GHQ-12) [14]; Yale single item (“Do you often feel sad or depressed?”) [15]; Depression Intensity Scale Circles (DISCs) [16]), communication (Frenchay Aphasia Screening Test (FAST) [17]), functional dependence (Barthel) [18], and locus of control (Recovery Locus of Control Scale) [19].

### Randomisation

Randomisation, stratified by the participants’ response to the Yale single-item question, was to MI or AC in a 1:1 ratio. Randomisation was conducted using opaque sealed envelopes. The envelopes were set up in shuffled blocks of four, with each block containing two allocations each to the MI or the AC arms. Therapist allocation (one of three therapists for each group) was carried out using opaque sealed envelopes. For each of the MI and AC groups, the envelopes were set up in blocks of nine, which contained three allocations for each therapist. These allocations were structured in a pseudorandom fashion so that no therapist’s workload would exceed six cases per week. Once a participant had consented and had their baseline data collected, the research nurse telephoned the research team administrator, informing them of the participant’s response to the Yale. The administrator then carried out the allocation process, firstly selecting an envelope to allocate the patient to a group, then based on group an envelope was selected for allocation to a therapist. The administrator then informed the research nurse of group and therapist allocation.

### Intervention delivery

The allocated intervention (MI or AC) was delivered face-to-face by the same therapist/visitor, in hospital or in the participant’s home. All sessions were audio recorded to allow therapists/visitors to reflect on and prepare for sessions, and to check consistency of technique. At the end of each session, therapists/visitors recorded the location, duration, and overall content of sessions on session forms developed for the study.

### Outcome measures

Outcome measures were collected via postal questionnaire at 3 months post-stroke, as the primary outcome in an effectiveness trial would be at this timepoint. Outcome measures included mood (GHQ12 [14]; Yale single item [15]; DISCs [16]), function (Barthel [18]; Nottingham Extended Activities of Daily Living Index [20]), quality of life (EQ-5D [21]), adjustment (Cognitive and Instrumental Readjustment [22]), and community integration (Community Integration Questionnaire [23]); a resource-use (health and social care input) questionnaire was sent out 2 weeks after the outcome measures questionnaire was sent out. If no outcome-measures questionnaire was returned within 4 weeks, and/or if no resourse-use questionnaire was returned within 2 weeks, a researcher (who was potentially non-blinded as they were involved in the randomisation process) contacted the participant by telephone as a prompt to complete the questionnaire. An unreturned questionnaire resulted in at least one prompting telephone call; a judgement as to whether further calls were made was based on a case-by-case basis, depending on the response to the first answered call.

### Study measures

Recruitment and reasons for exclusion or declining (if offered by the patient) were documented using screening logs. Randomisation and allocation to arm and therapist was documented on a randomisation log. Acceptability of the study measures was assessed by summarising the level of item missing data on returned questionnaires and through interviews with participants. Acceptability of therapist study measures was assessed by summarising the completion of the WAI by the MI therapists. Acceptability of the MI and AC was assessed through semi-structured interviews with staff and participants. Fidelity to the MI intervention (and MI manual) was monitored through review of audio-recordings of sessions using the MITI global ratings [10]. Fidelity to the AC intervention was monitored through review of audio-recordings and visitor session notes.

### Staff interviews

The MI therapists, AC visitors, therapy manager, and the research nurse involved in the screening and recruitment of patients were invited to be interviewed and gave their consent. A member of the research team conducted the interviews which explored staff perceptions of the study including their views on the acceptability and suitability of the MI, AC, study materials and training, the use of clinical staff as therapists/visitors, and the factors influencing the implementation of study processes. Staff were approached to participate in the interviews at the end of the study apart from two MI/AC staff who left their post during the study period. These staff were interviewed during the study while their involvement in the study was ongoing. The interviews with the intervention staff were conducted over the telephone; the interviews with the therapy manager and research nurse were conducted face-to-face. All interviews were digitally audio-recorded and transcribed verbatim.

### Participant interviews

Once follow-up was completed, two participants from the AC arm and two participants from the MI arm were randomly selected and invited to take part in semi-structured interviews with a member of the research team. Participants consented to the interviews which explored the acceptability of the interventions and study processes. Four key aspects were explored: (i) recruitment to the study, (ii) acceptability of the intervention received, (iii) suggested future improvements to the intervention, and (iv) study paperwork.

### Analysis

Descriptive statistics were used for patient eligibility, recruitment, allocation and retention, demographic and clinical characteristics, level of missing data for the study measures, and staff recruitment and retention. The analyses were conducted in SPSS, v21 and v22.

Content analysis was undertaken on the staff interviews by two researchers, using qualitative data analysis software (NVivo 10). To ensure reliability, a sample of the interviews was coded by both researchers independently. There was a good level of agreement between the two researchers; any differences in coding were discussed until a consensus was reached. Interpretation of the codes was carried out using the Consolidated Framework for Implementation Research (CFIR) [24] which is a taxonomy of factors that influence implementation. The CFIR framework consists of five key domains (intervention characteristics, inner setting, outer setting, characteristics of individuals, and process), with each domain containing sub-constructs. The framework attempts to explain the complex and often interacting factors which may influence implementation. The framework combines key concepts of implementation proposed across a number of previous models of implementation, seeking to integrate and consolidate the varying concepts into one framework. We used the CFIR for the interpretation of interview data to understand factors that influenced the implementation of the study.

One-fifth (*n* = 12) of the voice files for MI sessions were purposively selected to maximise a range of MI therapists, session number, time point during the study, participant sex, age, and baseline Yale for assessment of fidelity to MI. Two researchers independently listened to each voice file and rated it on the five global dimensions of the MITI: evocation, collaboration, autonomy/support, direction, and empathy, on a five-point scale. The average of the evocation, collaboration, and autonomy/support ratings creates an overall MI spirit rating. A higher rating indicates greater fidelity to MI. Any large discrepancies (difference of 2 or more points on the scale) in ratings were discussed until consensus reached or adjudicated by a third researcher.

Voice files of (*n* = 12) AC sessions were purposively selected to maximise a range of AC visitors, participant sex, age, and baseline Yale. Two researchers independently listened to each voice file to ensure there was no therapeutic content within the conversation.

## Results

### Recruitment and retention of staff

Six therapy assistants were recruited to undertake the study role of either MI therapist (*n* = 3) or AC visitor (*n* = 3) before participant recruitment commenced. Two MI therapists and two AC visitors left their clinical post, and therefore their study role, during the study and were not replaced. One AC visitor changed their clinical role moving to another clinical department, but continued in their study role.

### Participant recruitment and characteristics

**Fig. 1:**
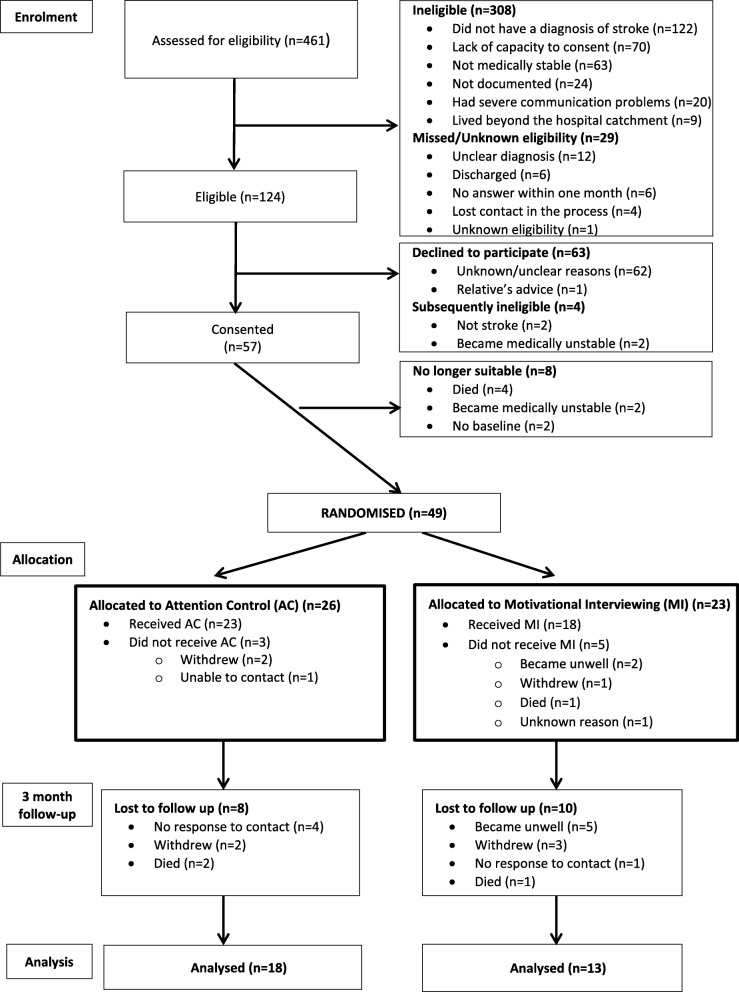
Eligibility, recruitment, and retention of participants

Participants were recruited during a 12-month period (December 2012 to November 2013). The flow of patients through the study can be seen in Fig. 1. Following screening, a low proportion (27%) of patients admitted to the acute stroke unit were screened as eligible for the trial. Of the 124 who were screened eligible, 57 (46%) consented to participate and 67 (54%) (95% CI 45 to 63%) declined to participate or became subsequently ineligible (reasons included relative’s advice, not stroke, became medically unstable). Of the 57, 8 (14%) were not randomised: 2 became medically unstable, 2 withdrew, and 4 died.

Forty-nine participants were randomised, 26 to the AC group and 23 to the MI group. The median (interquartile range [IQR]) age of the 49 participants was 71.0 (60.5–78.0) years and 27 (55%) were male. Twenty-three (47%) and 18 (37%) participants had left- and right-sided strokes respectively, with the remainder having bilateral (2, 4%) or no-sided (6, 12%) weakness.

At baseline, 47 (96%) of GHQ-12 questionnaires were completed indicating that 27 (57%) participants had abnormal values (> = 2). The ACE-R was completed by 46 (94%) participants, with 21 (46%) showing abnormal cognition (< 82).^1^ The FAST was completed by 45 (92%) participants, with 16 (36%) having abnormal communication (<= 27 for age 20 to 60, <= 25 for age 61+). Table 1 shows the baseline characteristics of participants by group.

**Table 1:** Baseline demographic and clinical characteristics for each group. Values are *n* (%) unless otherwise stated

	Attention Control (*n* = 26)^1^	Motivational interviewing (*n* = 23)^1^
Male	15 (58)	12 (52)
Age in years (median (range))	72.0 (43–91)	70.0 (28–88)
Depression:		
Yale: yes	8 (31)	8 (35)
GHQ-12:	*n* = 25	*n* = 22
Total score^2^ (mean (SD))	3.16 (3.80)	4.00 (3.77)
Abnormal mood (> = 2)	13 (52)	14 (64)
Stroke side:		
Left	10 (39)	13 (57)
Right	9 (35)	9 (39)
Bilateral	2 (8)	0 (0)
Neither	5 (19)	1 (4)
Abnormal cognition (cut-off < 82)	*n* = 24 11 (46)	*n* = 22 10 (45)
Abnormal cognition (cut-off < 88)	*n* = 24 15 (63)	*n* = 22 12 (55)
Abnormal communication	*n* = 24 6 (25)	*n* = 21 10 (48)
Physical function	*n* = 25	*n* = 22
Good	18 (72)	14 (64)
Moderate	4 (16)	2 (9)
Poor	3 (12)	6 (27)

^1^Total sample size applicable, unless otherwise stated

^2^Scores range from 0 to 12; higher scores indicate higher levels of depression

### Baseline measures

Of the data collected at the time of screening, the past/current psychological input and antidepressant use questions were completed for only 44 (90%) and 46 (94%) participants respectively.

Of the baseline assessments, there was 94% completion of most of the ACE-R items, and 92% completion of almost all FAST items. Items with lower completion rates across participants are shown in Table 2.

**Table 2:** Completeness of baseline items investigated through notes and performance-based tests for the total group

Measure/question	Baseline
	Total (*n* = 49)
Screening variables	
Age	49 (100%)
Sex	49 (100%)
Stroke side	49 (100%)
Past/current psychological input	44 (90%)
Antidepressant use	46 (94%)
Addenbrooke’s Cognitive Examination (ACE-R)	
Language writing: sentence	44 (90%)
Visuospatial abilities: clock	45 (92%)
Recognition	43 (88%)
All other 23 items	46 (94%)
Frenchay Aphasia Screening Test (FAST)	
Write score	44 (90%)
All other 13 items	45 (92%)

### Intervention delivery

Of the 49 participants randomised, 41 (84%) received their allocated intervention (at least 1 session completed); 23 (88%) of those allocated AC; 18 (78%) of those allocated MI. Thirty-one (76%) of the 41 had the maximum four sessions: 12 (67%) in the MI group, and 19 (83%) in the AC group.

A comparison between the first half and second half of participants recruited was conducted to investigate whether staff attrition impacted on the number of sessions delivered in each group. Participants randomised to AC who were among the first half recruited to the study all received at least one session, whereas three participants among the second half recruited received no AC sessions. There were also more participants receiving no MI sessions among the second half of participants recruited to the study compared to the first half. Fewer participants received four sessions of AC and MI among the second half recruited to the study compared to the first half; for the MI group, this was 50% fewer. The mean number of sessions received were 3.54 (AC) and 2.92 (MI) for those among the first half recruited, and 2.85 (AC) and 2.00 (MI) for those among the second half recruited.

The delivery of MI or AC tended to start between 2 and 7 weeks post-stroke for most participants; the median (IQR) time was 20 (15–47) days. MI and AC sessions were delivered over a mean of 3.6 weeks and a maximum of 10 weeks.

MI therapists and AC visitors completed documentation for 66% (MI) and 65% (AC) of the total sessions held. The WAI was completed for 10 (43%) of the MI participants. Only two of the three MI therapists completed the WAI on some occasions; one MI therapist did not complete the WAI on any occasion.

### Retention

At 3 months, 3/49 (6%) participants had died. Of those 46 participants recruited and not known to have died, 5 (11%) did not respond to contact, 5 (11%) had withdrawn, 5 (11%) could not respond due to being unwell, and 31 (67%) returned the questionnaire (Fig. 1).

We received 3-month follow-up data for 31/49 (63%) participants. Twenty-three participants returned their questionnaire by post without prompting. A further eight returned their questionnaire following one (*n* = 5), two (*n* = 1), or three (*n* = 2) telephone calls.

### Outcome measures—3 months

At 3 months, the completion of items within questionnaires was generally high. A large majority of the questionnaires were completed by the participant 27/31 (87%), 2/31 (6%) were completed by a relative and 2/31 (6%) did not respond to the question about who was completing the questionnaire. All outcome measures other than the Yale single-item had less than 100% completion (minimum 84%), although this was generally due to three respondents consistently not answering several items.

Descriptive statistics for the main outcome measures are shown in Table 3.

**Table 3:** Descriptive statistics for the main outcome measures at 3 months for each group. Values are *n* (%) unless otherwise stated

	Attention control (*n* = 18)	Motivational interviewing (*n* = 13)
Depression		
Yale: yes	5 (28)	5 (39)
GHQ-12		
Total score^1^ (mean (SD))	2.06 (3.69)	2.92 (4.13)
Abnormal mood (> = 2)	7 (39)	5 (39)

^1^Scores range from 0 to 12; higher scores indicate higher levels of depression

### Fidelity to MI

Due to study staff attrition, it was not possible to purposively sample based on the time point during the study; however, the voice files selected did cover a range of time points over the course of the study. In the 12 sessions reviewed, global ratings ranged between 3 and 5, indicating proficient to competent delivery of MI. Raters agreed on 42 out of all 60 ratings (70%), and where there were discrepancies, there was a difference of only one point on the rating scale.

### Fidelity to AC

In the 12 sessions reviewed, there were no instances of conversation that were considered therapeutic or similar to MI. Occasions of discussing mood and well-being were minimal, with three instances identified. AC visitors used strategies to avoid such discussions from becoming therapeutic, such as diverting the conversation to a neutral topic. This is described in more detail in a separate article (in preparation).

### Staff interviews

Seven staff interviews were conducted. These staff comprised the five therapy assistants who delivered the MI intervention or AC comparator to patients (three MI therapists; two AC visitors, one of whom went on to screen patients), the therapy team leader who manages the therapy assistants, and the research nurse who was involved in the screening and recruitment of patients.

Findings are presented and grouped into the four domains of the CFIR that were represented in the interview data: (1) intervention characteristics, (2) characteristics of individuals, (3) inner setting, and (4) process. The ‘outer setting’ domain of the CFIR was not represented in the interview data. A summary of key facilitators and barriers to conducting the study is presented in Tables 4 and 5.

**Table 4:** Barriers to conducting the study described using the CFIR

CFIR domain	Element	Barrier	Quote
Intervention characteristics	Design quality and packaging	Possibility of being allocated to attention control	“Patients did not cite AC as a reason for not participating in the study but personally feel it was an issue”
		Patients viewed intervention as burdensome	“One of the main reasons for people declining was essentially people were saying they have got enough on their plate”
		Baseline assessments were considered lengthy	“I do not sometimes feel like I can do a session after the baseline, sometimes they are tired”
Characteristics of individuals	Self-efficacy	Patients declining to participate reduced confidence to recruit	“This trial got the most negative responses…it sort of knocked my confidence a bit”
		MI therapists lacked confidence in their ability to deliver MI	“Do not feel a hundred per cent confident in my skills in MI, it’s difficult to know whether I am doing it right”
		MI skills weakened due to irregular recruitment	“There were periods with no patients so not doing MI, so felt I was losing skills a little bit”
	Other personal attributes	High turnover among therapy assistants	“Therapy assistants are looking for other jobs and there is high turnover among them”
Inner setting	Networks and communication	Lack of co-ordination for the trial on-site	“Would be better to have someone identified as the co-ordinator within the hospital, it was difficult knowing who was doing what”
	Structural characteristics	Backfill for therapy assistants was not always appropriate	“Backfill does not really cover my time… because of the way our team is made up… therapy assistants are not generic, we are specialised, so backfill was not appropriate”
	Available resources	Therapy assistants left their role	“Going from three therapists in each arm to one has been difficult”
	Leadership engagement	Supervisors lacked knowledge of the study	“Supervisors in [new department] did not know anything about the study so it has been a bit tricky to do the study role”
	Relative priority	Therapy assistant role prioritised over study role	“It’s difficult to say, ‘Oh no I cannot do that because I have got the motivational interviewing’….In some ways you feel like that should take priority over the MI”
Process	Executing	Not enough training and feedback	“Training was quite intensive to start with but then fizzled out when recruitment started… we did not get a lot of feedback… Would be useful to be able to refresh skills”

**Table 5:** Facilitators for conducting the study described using the CFIR

CFIR domain	Element	Facilitator	Quote
Intervention characteristics	Relative advantage	Intervention seen as beneficial to patients	“The patients have got somebody to talk to who’s neutral, they are not going to talk to their family because they do not want to worry them”
	Design quality and packaging	Delivering sessions weekly	“Sessions being once a week works well to help maintain rapport”
		Holding AC sessions in patients’ home	“Sessions at home were easier because there’s lots of pictures and postcards, you can be more natural asking questions”
Characteristics of individuals	Self-efficacy	Existing skills and previous experience	“Used to emotional aspects and sensitive issues from working with patients on the wards so able to deal with these”
		Confidence increased with experience during the study	“More comfortable with patients now than I was when we started with the practice patients”
Inner setting	Relative priority	Value of study	“Psychological services within stroke is very important and is often overlooked and I think that any form of research which looks into that and raises the awareness of that is good”
Process	Executing	Supervision from study team	“Supervisors have been very good… have found it useful to be able to email and ask what to do if unsure about things”

#### Intervention characteristics

The design quality and packaging of the AC and MI intervention was deemed an important factor for recruitment to the study. The possibility of patients receiving the AC rather than MI was perceived by screening staff as a potential barrier to recruitment. Screening staff also cited the onerous (as perceived by patients) nature of the AC and MI as a barrier to recruitment. However, the format of the MI intervention and the AC were generally viewed positively. Therapy assistants felt that weekly sessions were appropriate for building and maintaining rapport with patients. Going to patients’ homes to conduct sessions was time-consuming, but therapists felt comfortable doing this, and AC visitors in particular found sessions in patients’ homes easier as there were more cues available to facilitate a more natural conversation. Some therapists felt that an hour per session was too long, particularly in the first two sessions, where patients might experience fatigue.

#### Characteristics of individuals (therapy assistants)

Self-efficacy was an important factor in recruiting and in delivering MI and AC. Patients declining to participate impacted on recruiting staff’s confidence. Therapy assistants found some of the specific skills involved in delivering MI and AC difficult as these were not consistent with their natural style of conversation. Therapy assistants were generally comfortable dealing with patients’ emotional responses due to their previous experiences with patients, but felt daunted about managing the emotional responses they perceived they were not trained to manage. MI therapists were not confident about their MI skills. More generally, the therapy assistants reported difficulty with keeping to the topic and style of conversation relevant to their MI/AC session. Overall, therapy assistants felt that their skills and techniques improved through the course of the study as they gained more experience with more patients. However, they felt that their skills decreased during periods when they were not delivering sessions due to irregular patient recruitment.

#### Inner setting

The main barriers to conducting the study were related to the inner setting factors of ‘structural characteristics’ and ‘available resources’ and were difficulties associated with therapy assistants being in a dual role (as therapy assistant and their study role as either MI therapist or AC visitor). The therapy assistants found it difficult to balance both roles in terms of time and workload. Backfill was available, but as the therapy team was organised such that therapy assistants were specialised and not generic, the available backfill was not always appropriate. There was also staff attrition, with therapy assistants leaving their role and not being immediately replaced, resulting in increased workloads for the remaining therapy assistants, impacting on the time available to fulfil their study role. The therapy team leader suggested that it would be more efficient to have staff from higher bands undertaking the study roles of MI therapists and AC visitors as they are a less transient workforce.

#### Process

Therapy assistants found the training useful and highlighted the opportunity to practice with patients as the most beneficial aspect. MI therapists felt that it would have been useful to have had more, and continued, feedback from study supervisors once they started delivering MI to patients in the study. They also felt they would have benefitted from refresher training sessions throughout the study period to keep skills updated.

### Participant interviews

Four participants were interviewed, two from the MI group and two from the AC group. Key findings for each aspect explored are described in turn.

#### Recruitment to the study

A common factor which influenced participants’ decision to participate was the thought that they may help others in the future. One participant explained:

“They explained to me exactly what would be required and … I said straight away yes … if what I’m doing is helping other people then that’s great, that’s what it’s all about” (Participant 1)

#### Acceptability of MI sessions

Those receiving MI had both positive and negative experiences of the MI intervention. Taking part in MI sessions was felt to have been beneficial:

“[The MI therapist] used to sort of bolster you up a bit and make you feel, you know, enthusiastic” (Participant 2)

Participants felt the MI sessions had been positive and had met their expectations:

“An awareness of what to expect really and why … I’ve got these troubles and how you can overcome it so it was very informative. It’s what I expected, what they said to be honest” (Participant 3)

While one participant felt the MI sessions had been positive, they also explained that it was draining to talk about such an emotional subject and described their relief on ending the study:

“Interviewer: How did you feel when the process was over? Participant: Well sort of a sigh of relief really, I think, that it was over with” (Participant 2)

#### Suggested future improvements to the MI intervention

One participant felt more MI sessions would have been beneficial and highlighted their desire for group support:

“I thought they should have done more of it to be honest to have that extra initial bit it’d be good to keep going on that. I think the interviews were good but I thought that they’re not really long … I would like instead of one every so many weeks maybe one a week and a group discussion would have been good” (Participant 3)

In addition, one participant felt that ongoing support following the MI sessions would have been useful in order to provide reassurance:

“When the process is over it’s as if … you’re left alone. There’s no one there to fall back … like oh well I’ve just got to go ahead now, back to normal now is it, … but it’s adaptation after that, which is difficult. So a bit of support on that would have been great, just ongoing” (Participant 3)

#### Acceptability of AC sessions

Participants who engaged in the AC sessions had mixed responses about their experiences. One participant found engaging in sessions was a positive experience:

“[AC visitor name] was absolutely brilliant … very caring and … it’s like a daily diary really what I’ve been doing” (Participant 1)

One participant described feeling sad when AC sessions had finished because they had enjoyed them. However, another AC participant found the sessions were unhelpful and added to their feelings of stress. They stated

“I just found it rather stressful, I mean I dreaded her coming the second time” (Participant 4)

This participant was unsure of what to expect from the AC sessions, and a lack of familiarity with the AC visitor seems to have amplified their difficulty directing the conversation. The participant also felt that the AC visitor lacked initiation with conversation:

“I didn’t really see the point in the [AC sessions] at the time ... I’m a very self-contained person and I don’t really need company. [AC visitor] just came in and sat there and I felt well ‘what sort of a conversation does she want?’ … I find it difficult to make conversation with someone that is totally alien to me … She found it difficult to make conversation” (Participant 4)

#### Suggested future improvements to the AC intervention

One suggestion was that AC visitors should be in a position to initiate conversation and that the emphasis for this should not be left to the participant:

“I think they’ve got to be interested enough to bring up a conversation to draw people out” (Participant 4)

#### Study paperwork

Some participants found the baseline and follow-up questionnaires acceptable:

“I do admin work anyway so I’m used to all the admin stuff … that doesn’t bother me at all” (Participant 3)

However, one participant found completing the questionnaires a negative experience, stating

“I found it repetitive and rather a lot of it” (Participant 4)

Overall, some participants enjoyed the MI and AC sessions and would have liked to have received more, while others found the sessions challenging. Some participants found MI sessions beneficial but tiring, and some found AC sessions difficult; this may have been due to their own personality or the characteristics of the AC visitor.

## Discussion

In this feasibility study of MI post-stroke, the intervention was successfully delivered using members of the clinical team, and we achieved delivery of an attention control. We learned about changes that would be necessary to conduct a future trial. Here, we make recommendations for a future trial based on our results, rather than using prespecified criteria.

The recruitment rate in the original trial of MI post-stroke (59%) [8] was used to calculate the recruitment target for the current feasibility study. However, the target was not met, and the recruitment rate in the current study was 46%. Participant interviews suggest those who took part were motivated by the desire to help future patients. Barriers to achieving the recruitment target included a lower than expected number of eligible patients consenting to participate, and staff undertaking the screening and consent process lacking confidence. We had also assumed that the randomisation rate (% of those eligible who consented and were randomised) would be the same as the consent rate (% of those eligible who consented) as it had been in the original trial, but this was not the case: the randomisation rate in this study was 40%. This was possibly due to the randomisation being performed less quickly than in the original trial so there was more potential for participants to be lost prior to randomisation. There was also a smaller than expected number of patients being eligible for the study (27% of all patients screened), whereas the original trial had a higher eligibility rate (50%). However, these figures may not be directly comparable as the original trial included all patients with suspected stroke without a confirmed diagnosis due to the diagnostic pathway at the time, whereas in this study a confirmed stroke diagnosis was possible relatively soon after admission and was necessary for recruitment. Furthermore, despite our study protocol stating that all patients admitted to the acute stroke unit with suspected stroke should be screened, the screening staff screened all patients admitted to the acute stroke unit, not limited to those with suspected stroke and it is unclear exactly how many patients were admitted with suspected stroke. Therefore, the eligibility rate of 27% might not truly reflect the numbers eligible based on our criteria. Despite this, even if we were to exclude those patients without suspected stroke from the number assessed for eligibility, the eligibility rate in this study would be 37%. For a future trial, a more conservative estimate of eligibility (37%) and a 40% recruitment rate (% of those eligible that were randomised) will be used. Additionally, appropriate training and ongoing support will be provided to staff conducting screening and obtaining consent.

Overall retention of participants to 3 months was only 63%. Even including those who died among those with primary outcome data, as they were known to have a ‘poor outcome’, ‘retention’ was only 69%, which is still lower than in the original trial of MI post-stroke (86%) [8]. There were also several participants who were unable to be contacted or became unwell. In a future trial, further strategies will be implemented to increase the retention rate and completeness of follow-up data, based on the current evidence [25], including obtaining alternative contact details (e.g. participant’s relative) in addition to participant details to increase the likelihood of maintaining contact with participants. Key questions around the primary outcome measure that could be answered by the alternative contact might also increase the availability of outcome data. Incentives for completing postal questionnaires (e.g. pen included with postal questionnaire), as previously shown to be successful, might also increase response rate [26]. However, completion of individual questions by those participants who returned the questionnaire was good. The study paperwork was acceptable to most participants, although one participant felt there was too much paperwork to complete, with too much repetition. In a future study, there will be careful consideration of which measures are included in follow-up questionnaires to ensure minimal burden to participants while collecting adequate study data. Patient and public involvement during the design of a future trial will play an important part in informing the potential burden and acceptability of follow-up questionnaires.

MI therapists and AC visitors had been expected to complete documentation relating to the sessions they conducted. However, study staff documentation was not well completed, which could have implications for the quality of the delivery of the MI or AC. The MI intervention involved the therapist reflecting on sessions using session notes in order to prepare for subsequent sessions and maintain continuity. This was also incorporated into the AC to enable AC visitors to recall the content of previous sessions and maintain rapport with participants. Without session notes, the quality and content of the MI and AC may be compromised. It was also intended that MI therapists completed a measure of therapeutic alliance after each session which were to be collected once all sessions were completed. It was not feasible to perform the planned evaluation of therapeutic alliance as this documentation was only completed occasionally by two of the three MI therapists. In a future trial, training for study staff will emphasise the importance of completing study documentation and there should be closer and ongoing monitoring of documentation completion throughout the study by the research team. In a future multi-centre trial, study staff will be asked to complete and submit documentation electronically to the research team following each session they complete with participants.

It was possible to implement an attention control in our study. The AC intervention was acceptable to some but not all participants. Barriers included a lack of familiarity with the AC visitor and the AC visitor lacking initiation in conversation. This occurred despite the AC visitors being trained to use various strategies to maintain non-emotive conversations including completing crosswords, playing cards, or discussing current affairs with participants. It is unclear if difficulties were due to the personality of the participant or the characteristics of the AC visitor. In a future trial, the training for AC visitors will have more emphasis on using strategies to initiate conversation. Furthermore, AC visitors will be selected based on characteristics likely to be more conducive to carrying out the AC following the development of a person specification for an AC visitor.

Sufficient numbers of staff were recruited and trained to MI proficiency, so it is possible to have members of the clinical team deliver the intervention. MI therapists felt they would have benefitted from ongoing and refresher training which could be incorporated for a future trial. However, the feasibility of using clinical staff to undertake the study roles of MI therapist and AC visitor remains uncertain. The attrition rate of study staff was very high, mainly due to the nature of their clinical role as therapy assistants who are a very transient workforce. This is not an issue specific to our study site, so in a future multi-centre trial it might be more efficient to recruit and train staff at a higher band (e.g. therapists), who are likely to be less transient than therapy assistants, to undertake the study roles.

Study staff experienced difficulties fulfilling both their clinical and study roles during the study period, due to the increased workload. MI and AC sessions were intended to be delivered once every week; however, due to study staff capacity, there were some instances where there were longer periods in between sessions. Difficulties were compounded by inadequate backfill and study staff attrition. There were fewer participants completing the maximum four sessions among the second half of participants recruited, and this was more pronounced in the MI group, suggesting that staff attrition may have impacted on the dose received by participants. In a future trial, recruiting and training more staff per site might alleviate the resource issues of staff being in a dual role. However, this might have implications for the preservation of MI skills, as more therapists would mean lighter caseloads and therefore less opportunity to practice MI, highlighting the importance of replacing study staff swiftly after any departures.

Those receiving the MI intervention found it to be acceptable, with some patients suggesting more sessions would be desirable. The need for ongoing support following MI sessions, including group support, was highlighted as something which may alleviate feelings of isolation and may support patients to cope in the longer term. In a future study, contact details for local groups will be provided to participants after follow-up.

The MI and AC interventions were also generally deemed acceptable by the MI therapists and AC visitors. However, the interviews with the staff undertaking these study roles were conducted by members of the research team and so the staff might have been inhibited in their responses. Additionally, due to resource issues, only four participant interviews were conducted limiting their generalisability.

## Conclusions

Our feasibility study showed that it is possible to train clinical staff to deliver MI, and an appropriate AC can be implemented. Although this feasibility study was conducted in only one centre, and some issues may be specific to the study site, we were able to identify changes to the study design and its implementation that would be necessary for a future multi-centre trial. We recommend the following changes for a research team to consider for conducting a future trial: Using a more conservative recruitment rate estimate than that used for the current study, implementing more strategies to increase participant retention, having therapists undertake study staff roles, and monitoring them on an ongoing basis. These changes would make it more feasible to conduct a multi-centre effectiveness trial of MI post-stroke, although some, such as the impact of the revised recruitment rate on achieving target sample size, may merit including an internal pilot in the design of the trial.

## Abbreviations

AC: Attention control
ACE-R: Addenbrooke’s Cognitive Examination Revised
ACTNoW: Accessing Communication Therapy in the North West study
CFIR: Consolidated Framework for Implementation Research
DISCs: Depression Intensity Scale Circles
FAST: Frenchay Aphasia Screening Test
GHQ-12: General Health Questionnaire 12-item
IQR: Inter-quartile range
MI: Motivational interviewing
MITI: Motivational Interviewing Treatment Integrity code

## Footnotes

1 Thirty participants (65% of patients who completed the ACE-R questionnaire) had abnormal cognition based on scores < 88.

## References

[1] Adamson J, Beswick A, Ebrahim S (2004). Is stroke the most common cause of disability?. J Stroke Cerebrovasc Dis.

[2] Hackett M, Pickles K (2014). Part I: frequency of depression after stroke: an updated systematic review and meta-analysis of observational studies. Int J Stroke.

[3] Willey JZ, Disla N, Moon YP, Paik MC, Sacco RL, Boden-Albala B (2010). Early depressed mood after stroke predicts long-term disability: the northern Manhattan stroke study (NOMASS). Stroke.

[4] Bartoli Francesco, Lillia Nicoletta, Lax Annamaria, Crocamo Cristina, Mantero Vittorio, Carrà Giuseppe, Agostoni Elio, Clerici Massimo (2013). Depression after Stroke and Risk of Mortality: A Systematic Review and Meta-Analysis. Stroke Research and Treatment.

[5] Feeling overwhelmed. The Stroke Association, Summer 2013. https://democratic.trafford.gov.uk/documents/s1689/Item%209%20Feeling%20Overwhelmed%20-%20Emotional%20Impact%20of%20Stroke.pdf.

[6] Hackett ML, Anderson CS, House AO, Halteh C. Interventions for preventing depression after stroke. Cochrane Database Syst Rev. 2008. 10.1002/14651858.CD003689.pub3. 10.1002/14651858.CD003689.pub3 18646094

[7] Hackett ML, Anderson CS, House A, Xia J. Interventions for treating depression after stroke. Cochrane Database Syst Rev. 2008. 10.1002/14651858.CD003437.pub3. 10.1002/14651858.CD003437.pub3 18843644

[8] Watkins CL, Auton MF, Deans CF, Dickinson HA, Jack CI, Lightbody CE (2007). Motivational interviewing early after acute stroke: a randomized, controlled trial. Stroke.

[9] Watkins CL, Wathan JV, Leathley MJ, Auton MF, Deans CF, Dickinson HA (2011). The 12-month effects of early motivational interviewing after acute stroke: a randomized controlled trial. Stroke.

[10] Moyers TB, Martin T, Manuel JK, Miller WR, Ernst D. Revised global scales: motivational interviewing treatment integrity 3.1.1. Center on Alcoholism, Substance Abuse and Addictions (CASAA); 2010.

[11] Bowen A, Hesketh A, Patchick E, Young A, Davies L, Vail A (2012). Clinical effectiveness, cost-effectiveness and service users’ perceptions of early, well-resourced communication therapy following a stroke: a randomised controlled trial (the ACT noW study). Health Technol Assess.

[12] Horvath AO, Greenberg LS (1989). Development and validation of the Working Alliance Inventory. J Couns Psychol.

[13] Mioshi E, Dawson K, Mitchell J, Arnold R, Hodges JR (2006). The Addenbrooke’s cognitive examination revised (ACE-R): a brief cognitive test battery for dementia screening. Int J Geriatr Psychatry.

[14] Goldberg D (1992). General health questionnaire (GHQ-12).

[15] Watkins CL, Lightbody CE, Sutton CJ, Holcroft L, Jack CI, Dickinson HA (2007). Evaluation of a single-item screening tool for depression after stroke: a cohort study. Clin Rehabil.

[16] Turner-Stokes L, Kalmus M, Hirani D, Clegg F (2005). The Depression Intensity Scale Circles (DISCs): initial evaluation of a simple assessment tool for depression in the context of brain injury. J Neurol Neurosurg Psychiatry.

[17] Enderby PM, Wood VA, Wade DT, Hewer RL (1986). The Frenchay Aphasia Screening Test: a short, simple test for aphasia appropriate for non-specialists. Disabil Rehabil.

[18] Wade DT, Collin C (1988). The Barthel ADL Index: a standard measure of physical disability?. Int Disabil Stud.

[19] Partridge C, Johnston M (1989). Perceived control of recovery from physical disability: measurement and prediction. Br J Clin Psychol.

[20] Nouri FM, Lincoln NB (1987). An extended ADL scale for use with stroke patients. Clin Rehabil.

[21] EuroQol Group (1990). EuroQol: a new facility for the measurement of health-related quality of life. Health Policy.

[22] Ben Sira Z, Eliezer R (1990). The structure of readjustment after heart attack. Soc Sci Med.

[23] Willer B, Rosenthal M, Kreutzer S, Gordon A, Rempel R (1993). Assessment of community integration following rehabilitation for traumatic brain injury. J Head Trauma Rehabil.

[24] Damschroder LJ, Aron DC, Keith RE, Kirsh SR, Alexander JA, Lowery JC (2009). Fostering implementation of health services research findings into practice: a consolidated framework for advancing implementation science. Implement Sci.

[25] Abshire M, Dinglas VD, Cajita MIA, Eakin MN, Needham DM, Himmelfarb CD (2017). Participant retention practices in longitudinal clinical research studies with high retention rates. BMC Med Res Methodol.

[26] Bell K, Clark L, Fairhurst C, Mitchell N, Lenaghan E, Blacklock J, Cushnaghan J (2016). Enclosing a pen reduced time to response to questionnaire mailings. J Clin Epidemiol.

